# Study of the Mechanisms and Therapeutic Approaches of Migraine in Women and Pregnancy: A Literature Review

**DOI:** 10.7759/cureus.35284

**Published:** 2023-02-21

**Authors:** Jimmy Barus, Harvey Sudharta, Dini Adriani

**Affiliations:** 1 Neurology, Atma Jaya Catholic University of Indonesia, North Jakarta, IDN; 2 Neurology, Said Sukanto Hospital, Jakarta, IDN

**Keywords:** pathophysiology, mechanism, pregnancy, women, migraine, headache

## Abstract

Headache is a significant and debilitating health problem, affecting more than half of the population worldwide. Migraine is a type of headache that is strongly associated with women and accounts for the high number of years lived with disability among women. The pathophysiology of migraine attacks may begin with a premonitory phase, followed by an aura phase and migraine headache. In women, many factors influence the prevalence of migraine, and sex hormone fluctuations around the menstruation cycle were believed to impact the pathogenesis of migraine. The International Classification of Headache Disorders, 3rd edition identifies menstrual migraine as pure menstrual migraine without aura and menstrually related migraine without aura. While migraine without aura (MwoA) was clearly associated with menstruation, migraine with aura (MwA) was generally unrelated to menstruation. Studies suggested that estrogen withdrawal is a trigger for MwoA, but high estrogen states are a trigger for MwA. During pregnancy, the increase in estrogen hypothetically prevents migraine attacks. There are several strategies for managing menstrual migraine, from acute/abortive, mini-preventive, and continuous preventive treatment. Managing migraine during pregnancy follows a similar strategy, but the drugs’ safety profile should be considered.

## Introduction and background

Headache is a significant, most debilitating health problem worldwide, as seen in the data provided by the Global Burden of Disease (GBD). It ranked second as the disease with the most years lived with disability (YLD) right after back pain; headache had 6.87% of total YLDs ranging from 1.18% to 14.19% vs. back pain with 9.39% of total YLDs ranging from 7.75% to 11.13% [1]. A collection of epidemiological studies confirmed GBD data in finding that headache disorders remain highly prevalent, affecting 52.0% (95% CI: 48.9%-55.4%) of the population worldwide [2].

One type of primary headache that is strongly associated with women is migraine. It is a predominantly female disorder with a two-fold higher lifetime incidence (43% vs. 18%) and a three-fold higher one-year migraine prevalence (17% vs. 6%) compared with men [2-4]. The International Classification of Headache Disorders, 3rd edition (ICHD-III) described migraine as a recurrent headache disorder, characterized by unilateral, throbbing, and severe intensity pain. Although, some cases may be bilateral or diffuse. According to GBD data, migraine causes 47.2 million YLDs (95% CI: 30.0-68.7) and ranks second in YLD among all causes of disability worldwide. It was estimated that 1.3 (95% CI: 1.2-1.4) billion persons were affected, resulting in a global all-age point prevalence of 18% [1]. In Indonesia, research on migraine prevalence in the large population has not been widely carried out. Therefore, no valid data are available [5].

The ICHD-III identifies menstrual migraine as pure menstrual migraine without aura and menstrually related migraine without aura. Pure menstrual migraine without aura occurs exclusively on day 1 ± 2 (days -2 to +3) of menstruation in at least two out of three menstrual cycles at no other times of the cycle, whereas menstrually related migraine without aura may occur outside of this menstrual migraine window. Every endometrial bleeding resulting either from the normal menstrual cycle or from the withdrawal of exogenous progestogens (combined oral contraceptives or cyclical hormone replacement therapy) is considered menstruation [6]. Pure menstrual migraine is uncommon and accounts for only 10-20% of cases, in contrast to menstrually related migraine that affects more than 50% of women with migraine [7,8].

## Review

Pathophysiology of migraine

The development of migraine was correlated with the individual’s susceptibility threshold, the mechanisms that trigger the attack, and the associated symptoms. Vulnerability to migraine is inherited, with several genes linked to this: (1) four different missense mutations of the α1A subunit of the P/Q-type voltage-gated calcium channel (chromosome 19) that is responsible for familial hemiplegic migraine (FHM); (2) mutation in gene ATP1A2 (chromosome 1q23) that encodes alpha 2 subunits of Na+/K+ pump will disrupt ATP1A2 allele function; (3) variations within the dopamine D2 receptor gene [9].

Migraine attacks may begin with a premonitory phase, which begins with nonpainful symptoms occurring hours to days (as early as three days) before the onset of actual [10]. Several common symptoms during this phase include neck stiffness, yawning, fatigue, mood changes, food cravings, and photophobia. Involvement of several key brain structures like the hypothalamus, brainstem, limbic system, and certain other regions of the brain was suspected, as observed by neuroimaging studies [11,12]. This may explain the autonomic symptoms experienced during this phase. Two main theories are proposed as the trigger of these structures: meningeal nociceptors activation by the parasympathetic tone and the activation of pain signaling from the trigeminal nucleus caudalis (TNC) to supratentorial pain-processing structures [13]. The nociceptive pathways that increase parasympathetic tone may be caused by migraine triggers (such as stress, awakening, or emotional changes), although stress and emotional factors may also contribute to migraine attacks through other pathways. The release of norepinephrine and sympathetic outflow contribute to the increased activity of pain signaling through actions on dural afferents and dural fibroblasts. This causes the release of neuropeptide neurotransmitters from parasympathetic efferents, which innervate the meninges and blood arteries, resulting in peripheral nociceptor activation [14].

The following step, the aura phase, is based on Aristides Leao's 1944 cortical spreading depression (CSD) hypothesis. It is distinguished by a gradual (2 to 6 mm/min) spreading wave of depolarization in neuronal and glial cell membranes, followed by cortical inhibition. This inhibition is what gives rise to the aura symptoms felt in many migraine sufferers. CSD was also associated with a wave of hyperemia, followed by cortical oligemia. It started from a rapid depolarization and repolarization of hyperexcitable neurons (related to migraine vulnerability) in the cerebral cortex. Thus, resulting in local accumulation of extracellular potassium ion (K+) that will continue to depolarize surrounding and adjacent neurons chronically. This large efflux of K+ was associated with changes in ionic gradients and disruption in many physiological functions like cell membrane, glutamate release, and sodium (Na+) and calcium (Ca2+) influx. CSD also contributed to trigeminal nociceptor activation and headache triggers as observed in several animal studies [15-17].

**Figure 1:**
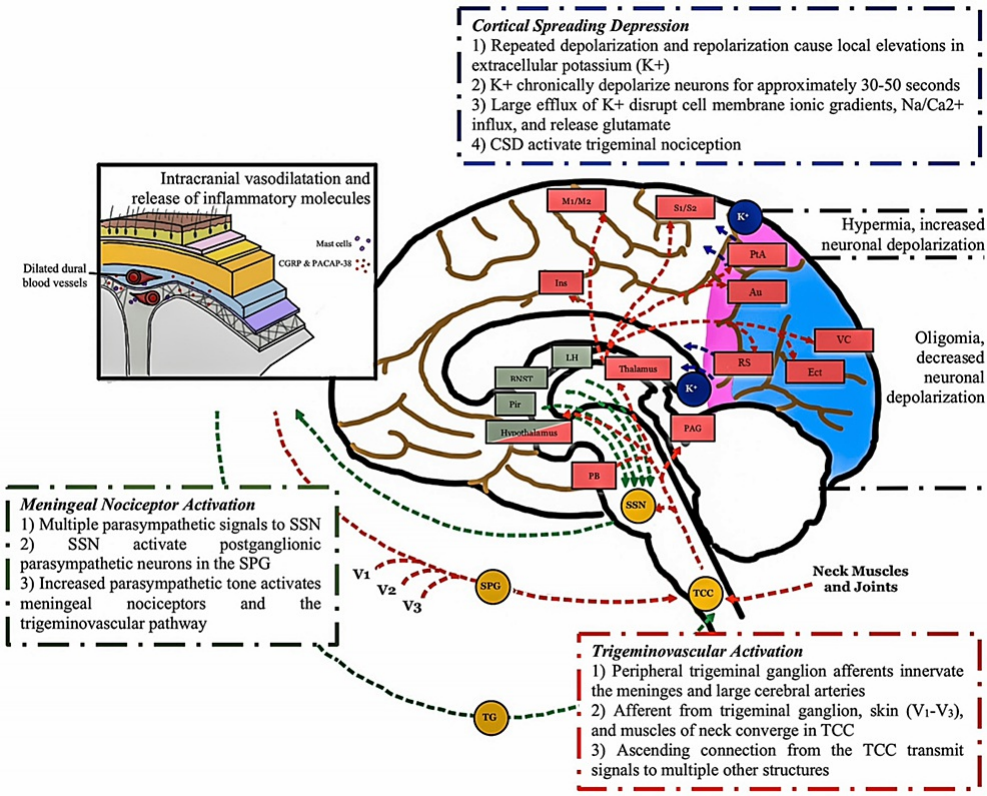
The mechanism of migraine Three hypotheses are believed as the mechanism of migraine attack: the cortical spreading depression (dark blue color), activation of meningeal nociceptor by the increase of parasympathetic activity (green color), and the trigeminovascular pathway (red color). Au = auditory; BNST = bed nucleus of stria terminalis; CSD = cortical spreading depression; Ect = ectorhinal; Ins = insula; LH = lateral hypothalamus; M1/M2 = motor cortices; PAG = periaqueductal gray; PB = parabrachial nucleus; Pir = piriform cortex; PtA = parietal association; RS = retrosplenial; S1/S2 = somatosensory cortices; SPG = sphenopalatine ganglion; SSN = superior salivatory nucleus; TCC = trigeminal cervical complex; TG = trigeminal ganglion; VC = visual cortices; V1 = ophthalmic branch of trigeminal nerve; V2 = maxillary branch of trigeminal nerve; V3 = mandibular branch of the trigeminal nerve. Figure illustrated by the author.

The migraine headache phase primarily involves the afferent neurons in the trigeminovascular pathway. It acts like a relay station to convey nociceptive information from the meninges to several parts of the brain, including the central and cortex areas. Nociceptive fibers from the trigeminal ganglion's ophthalmic branch innervate the meninges and major cerebral arteries [18]. Inputs from neighboring structures (skin, muscles, C1-C2 innervated tissues) merge into trigeminal ganglion afferent projections before synapsing on second-order neurons in the trigeminal cervical complex (TCC). These extracranial neurons are responsible for pain sensation in the periorbital, occipital, and cervical-neck areas. Signals will subsequently be transmitted to various other brain regions like the brainstem, thalamic, hypothalamic, and basal ganglia nuclei, through ascending pathways from the TCC, giving rise to several associated symptoms of migraine (phonophobia, photophobia, cognitive dysfunction, allodynia, and osmophobia) (Figure 1) [19,20].

Migraine and sex hormones

Migraines are more common in women than in men. Because menarche, menstruation, pregnancy, menopause, and hormonal contraceptives all had an effect on migraine frequency, it was assumed that sex hormones were involved. Several studies have found that migraines affect both sexes equally until puberty, and their frequency increases after menarche [21,22].

Changes in sex hormones such as estrogen and progesterone were thought to influence migraine pathophysiology. Migraine without aura (MwoA) was definitely correlated with menstruation, but migraine with aura (MwA) was not. Research shows estrogen withdrawal triggers MwoA, and excessive estrogen states trigger MwA [23]. Animal studies have confirmed differences with respect to sex hormones in migraine between male and female rats. In humans, research has indicated that the incidence of MwoA peaks during a five-day menstrual attack window that begins two days before the onset of menstruation and extends through the first three days of menstruation [7,23-25]. In a population-based study, Stewart et al. [25] noted an increase in MwoA attacks on the first two days of menstruation, and the number decreased around the expected time of ovulation. The duration appeared to be significantly longer for migraine headaches in the three to seven periods before the onset of menses. Most available studies agreed that migraine during the cycle is far worse than outside of the cycle in terms of duration, frequency, intensity, and disability [25,26]. Some studies also suggested that menstrual migraine attacks accompany nausea and vomiting more than non-menstrual attacks [23,27].

The pathophysiology of menstrual migraine was originally linked to estrogen withdrawal over 30 years ago. The finding that estradiol administered to female migraineurs could delay migraine attacks until the level dropped to pretreatment levels supported this notion [28]. Several biological conditions associated with a fall in estrogens (e.g., before menstruation, hysterectomized women, baby delivery, and pill-free period in women using oral hormone replacement therapy, receiving gonadotropin-releasing hormone [29]) have also repeatedly been shown to be associated with a worsening of migraine attacks. On the opposite, high estrogen levels (e.g., second and third trimester of pregnancy, continuous use of combination oral contraceptives, and menopause) were linked to protection against MwoA [30]. Irregular fluctuation of the circulating sex hormones during perimenopause was associated with worsening or change in migraine patterns [31].

The capacity of estrogens to interfere with cellular excitability or cerebral arteries may explain migraine attacks that occur outside of the low estrogen period. Passive diffusion allowed these hormones to permeate the blood-brain barrier, and their level in the blood was found to be equivalent to that of the central nervous system. Estrogen and progesterone could affect the pain-processing networks and endothelium implicated in migraine pathogenesis. Estrogens were discovered to influence brain neurotransmitters such as serotonin, dopamine, norepinephrine, and endorphins. It aided the glutaminergic system, which may have increased neuronal excitability. Not just hormones but prostaglandins have been linked to menstrual migraines. It is a hormone-like chemical that has been associated with throbbing headaches, nausea, and vomiting when it enters the systemic circulation. It seemed to activate GABAergic systems while suppressing neuronal response. The pathogenesis of migraine includes the induction of CSD, which is linked to glutaminergic transmission [32,33].

High estrogens may enhance women’s susceptibility to MwA by increasing cortical excitability. This was substantiated by the fact that MwA could occur while beginning a combination of estrogen-progestin oral contraception (COC), hormone replacement therapy, or during pregnancy. However, not every woman will experience migraine episodes during this time period. While other women are not, certain women's sensitivity to migraine attacks may be related to their capacity to metabolize estrogens or polymorphisms in genes coding for sex hormones, receptors, or metabolites of the hormonal pathways [34,35].

Migraine and pregnancy

During pregnancy, estrogen levels may rise to roughly one hundred times the normal level, and hormonal fluctuations are also more prominent. The increase in estrogen hypothetically prevented MwoA attacks. Most female migraineurs reported a decrease in migraine attack frequency from the first to the third trimester [31]. MwA may improve less during pregnancy. A study by Granella et al. [36] found that improvement in headaches during pregnancy only occurred in 43% of females with MwA, compared to 72% of patients with MwoA. Despite the fact that most pregnant women suffer fewer migraines, pregnancy itself is associated with headaches from secondary causes such as preeclampsia, stroke, and venous sinus thrombosis [26,27].

In practice, headache/migraine attacks in pregnant women who have no history of primary headaches or headaches that increase or change in quality should be treated as secondary headache syndrome until proven else. Several conditions, like gestational hypertension, preeclampsia, eclampsia, cerebral venous thrombosis, pituitary apoplexy, and idiopathic intracranial hypertension, may also cause headaches similar to a migraine attack. It is important to be able to identify “red flag symptoms” during pregnancy [27].

Treatment of migraine in women

Up to this date, there is no single medication/drug that has the US Food and Drug Administration (FDA) approval for the treatment of menstrual migraine or either of its subtypes, i.e., the pure menstrual migraine (PMM) and menstrually related migraine (MRM). However, multiple studies have been conducted, and certain medications seem effective. The American Migraine Foundation suggested several treatments for menstrual migraine, from acute abortive management to preventive management (mini-preventive or continuous preventive) [37]. The drugs and doses available for managing menstrual migraine are summarized in Table 1.

**Table 1:** Drugs and doses available for the treatment of menstrual migraine NSAID = nonsteroidal anti-inflammatory drugs; PO = per oral; SC = subcutaneous injection.

Drug class	Drug	Dose	Note
Abortive treatment			
Triptans	Rizatriptan	10 mg PO, single dose	Has the best overall evidence for pain freedom at two hours
	Sumatriptan	4-6 mg SC, single dose	The only injectables with a faster action onset (bypassing the digestive tract)
Mini prevention			
NSAID	Naproxen	550 mg PO, two times daily. Starting before the period, continue for five to seven days	If a migraine attack still occurs during consumption, usually, it is less severe and responsive to triptans
Supplement	Magnesium supplementation	360 mg elemental Mg PO, three times daily. Starting on the 15th day of the period, continue until the next menstrual cycle	No regular cycles are required to time this method
Triptans	Frovatriptan	2.5 mg PO, two times daily. Starting two days before the period, continue until three days after the period	For mini-preventive treatment, the American Headache Society Evidence-Based Guidelines assessed frovatriptan as effective (Class A) and naratriptan and zolmitriptan as possibly effective (Class B). The FDA did not issue a recommendation for triptan usage because they believe the evidence of benefit is insufficient
	Zolmitriptan	2.5 mg PO, 2-3 times daily. Starting two days before the period, continue until three days after the period	

Acute Treatment

Several acute treatments for managing menstrual migraines include fast-acting triptans, injectables, or nasal triptans. Oral fast-acting triptans (sumatriptan, zolmitriptan, almotriptan, rizatriptan, or eletriptan) [38-41] administered early in a migraine attack in combination with a nonsteroidal anti-inflammatory drug (NSAID) may be enough to manage the symptoms. It should be noted that these drugs are not to be used in contraindicated populations because of their high cardiovascular risks. Contraindications to triptans include coronary artery disease, a history of stroke, peripheral vascular disease, and uncontrolled/severe hypertension [42].

Mini-Preventive Treatment

When acute therapy is not sufficient to relieve symptoms or reduce disability, perimenstrual or continuous prophylaxis might be an option. It usually consists of taking medication prior to the onset of menstrual for five to seven days only. This method does not have FDA approval, but several studies have been conducted related to this. The choice of prophylaxis depends on many factors, like the type of migraine, regularity of menstruation, other health-related problems, and the need for contraception (Table 1) [43].

NSAIDs taken twice a day during the time period (five to seven days around the start of a period) may prevent or at least lessen the severity of the migraine attack. Magnesium has shown promising results in a controlled trial. Usually, it starts on day 15 of the menstrual cycle (or 15 days from the beginning of the period) and continues until the next period begins. Long-lasting triptans such as naratriptan and frovatriptan twice a day throughout a woman’s period also appeared to decrease or prevent menstrual migraine. Prolonged triptans use may cause medication overuse [43].

Several trials have been conducted to evaluate the efficacy of estrogen in gel [27,44] or patches [45,46] form to reduce estrogen fall during perimenstrual days. Following a similar technique, another open-label research found an oral contraceptive containing 20 mcg ethinyl estradiol on days one to 21, supplemented with 0.9 mg conjugated equine estrogens on days 22 to 28, was effective in preventing menstrual migraine. Short-term estrogen supplementation is successful as long as it prevents estrogen withdrawal; thus, the optimal time to utilize estrogen supplementation is during the entire estrogen-free interval of oral contraceptives, particularly if they contain low estrogen dosages. Low-dose estrogens and/or progestins (estradiol valerate plus dienogest) are an alternate technique for preventing menstrual migraine among women considering contraception [47].

Continuous Preventive Treatment

This approach is recommended for women who have irregular periods or for whom mini-prevention does not work. Continuous preventive treatment mostly targets hormones because the hormone drop precipitates a migraine headache. Dosing birth control pills continuously without a break (extended cycle) can effectively reduce menstrual migraine. Alternatively, monthly vaginal ring insertion with a break allowing menstrual period every three to six months (may use mini-prevention strategy at this period of time) may be recommended. In patients using other forms of hormonal contraception with estrogen, use the lowest possible dose to minimize the drop in estrogen during a pill-free week [35]. However, these hormonal medications have many side effects and should be reserved only for the most refractory patients [48].

Treatment of migraine in pregnancy

Conservative treatment has a beneficial effect on headaches in pregnancy. Several nonpharmacologic measures can be recommended, including optimizing sleep hours and sleep quality, eating regular meals, regular exercise, and staying hydrated [49]. Supplements like coenzyme Q10 (CoQ10) and magnesium also showed some benefits in pregnancy [50]. During pregnancy and breastfeeding, the preferred therapeutic strategy should always be a non-pharmacological one [37,51].

Pharmacologic therapy might be considered when conservative measures fail, judging from the risk/benefit ratio. According to the FDA, the majority of drugs were evaluated as C, making it difficult to use the label alone for clinical practice. If randomized trials are not available, clinical experience guidance and expert consensus can be employed [51]. Table 2 summarizes the most commonly used abortive and preventive drugs during pregnancy and breastfeeding.

**Table 2:** Medications for headaches during pregnancy and lactation. ADHD = attention deficit hyperactivity disorder.

Drug class	Drug (FDA category)	Use in pregnancy	Use in lactation
Abortive medications			
Analgesics	Acetaminophen (B)	Safe to use; possible risk of increased ADHD	Safe to use
	Ibuprofen (B), Naproxen (B)	Safe to use in the first and second trimesters; use in the third trimester is not advised as it may cause premature closure of the ductus arteriosus, postpartum hemorrhage, and neonatal bleeding	Safe to use
Antiemetic	Metoclopramide (B)	Safe to use if consumed intermittently; chronic use may be associated with dystonic reaction and should be avoided	Safe to use
	Ondansetron (B)		
Ergots	Ergotamine (X)	Contraindicated; risk of miscarriage (from uterine hypertonicity and vasoconstriction) and congenital defects	Not safe to use; cause nausea/vomiting, and suppresses prolactin secretion and lactation
Triptans	Sumatriptan (oral) (C)	May be considered for 1st and 2nd trimesters; was associated with spontaneous abortion	Safe to use
Steroids	Prednisone (C), Dexamethasone (C)	May be considered for severe migraine by occasionally using. Long-term use was linked to congenital anomalies, fetal/neonatal adrenal suppression, and stillbirth	Safe to use; avoid high doses because it may suppress lactation
Prophylactic medications			
Antiepileptic	Topiramate (D)	Not recommended; may cause cleft lip/palate	Safe to use; monitor for diarrhea and drowsiness
	Valproic acid (X)	Contraindicated; high risk of fetal abnormalities (teratogenic)	Safe to use
Beta-blockers	Propranolol (C), Metoprolol (C)	May be considered; may cause fetal bradycardia and uterine hypotonicity. Taper off a few days before delivery is recommended	Safe to use
Calcium-channel blockers	Verapamil (C)	May be considered; should be avoided in late pregnancy due to its tocolytic effect and risk of fetal hypoxia	Not recommended; excreted in human milk
Tricyclic antidepressants	Amitriptyline (C), Nortriptyline (C)	May be considered at a low dose (25–50 mg/d); no deleterious effects observed but may cause neonatal drowsiness. Taper off weeks before delivery is recommended	Safe to use

Abortive Medications

Analgesic to lessen the severity of headache consists of acetaminophen and NSAIDs. Acetaminophen up to 4 grams a day is considered safe and is widely used as the analgesic of choice in pregnancy (category B). NSAIDs like ibuprofen and naproxen are not to be consumed during the third trimester due to the risk of ductus arteriosus premature closure, postpartum hemorrhage, and neonatal bleeding. Antiemetics like metoclopramide (category B) and ondansetron (category B) are relatively safe to use. Metoclopramide has been widely used as it also has antinociceptive effects in migraine. Ergotamine is absolutely contraindicated because of the increased risk of miscarriage. Several congenital defects have been linked to this drug [52]. It is also not recommended during lactation as it suppresses prolactin secretion and causes nausea/vomiting [53]. Triptans may be considered, although their vasoconstrictor properties may carry risks during pregnancy. Several studies supported this finding that there is no increased risk of pregnancy outcomes following prenatal exposure to triptans [54,55]. Steroids may be considered, but their use is limited and not for the long-term because of the risk of congenital anomalies, fetal/neonatal adrenal suppression, and stillbirth. During breastfeeding, steroid use is considered safe, although it may decrease milk production if consumed at very high doses. Prednisone is the preferred steroid since it is converted by the placenta to an inactive form with minimal fetal harm [56].

Prophylactic Medications

Beta-blockers and magnesium are frequently used and remain the medicine of choice during pregnancy. It is advisable to taper/stop the medicine a few days before birth to limit the chances of fetal bradycardia and uterine contractions. Several adverse effects observed in some studies are intrauterine growth retardation, preterm birth, and respiratory distress [57,58].

Low-dose tricyclic antidepressants are considered safe to use during pregnancy, although their use was associated with an increased risk of preterm birth in infants [59]. High doses should be avoided because of their association with overall malformations, like limb deformities and heart defects. The lowest effective dosage should be prescribed to minimize adverse effects and taper off four weeks before delivery is recommended [60]. The use of venlafaxine, especially in late pregnancy, is associated with neonatal withdrawal syndrome (irritable, restless, hypertonic infant). Calcium channel blockers like verapamil are relatively safe in early pregnancy but should be avoided during late pregnancy as it has a tocolytic effect on the uterus. Because it is excreted in human milk, it has been linked to adverse consequences during lactation. Antiepileptic drug use is not advised because it is linked to an increased risk of cleft lip/palate. Valproate is not recommended because of the significant risk of fetal defects. However, it is safer to use during lactation [61].

## Conclusions

Migraine is a complex disease with various pathways arising from the overstimulation of the pain-sensitive structure in the brain. The pathophysiology of migraine can be explained in three main phases: the promontory, aura, and migraine headache phases. Women’s migraine attacks were related to hormonal changes, primarily caused by a low-estrogen period, affecting the cellular excitability and cerebral vessels, and causing MwoA. On the other hand, high estrogen levels may also trigger MwA by increasing cortical excitability. Managing menstrual migraine in women and in pregnancy follows a similar approach to migraine in general. The only difference is in the timing of consumption related to the menstrual cycle in women and the risk-to-benefit consideration in pregnancy.
